# In Vitro Transcriptome Response to a Mixture of *Lactobacilli* Strains in Intestinal Porcine Epithelial Cell Line

**DOI:** 10.3390/ijms19071923

**Published:** 2018-06-30

**Authors:** Ionelia Taranu, Daniela Eliza Marin, Cornelia Braicu, Gina Cecilia Pistol, Ionut Sorescu, Lavinia Lorena Pruteanu, Ioana Berindan Neagoe, Dan Cristian Vodnar

**Affiliations:** 1 Laboratory of Animal Biology, National Institute for Research and Development for Biology and Animal Nutrition, Calea Bucuresti No. 1, Balotesti, 077015 Ilfov, Romania; daniela.marin@ibna.ro (D.E.M.); gina.pistol@ibna.ro (G.C.P.); ionutsorescu68@gmail.com (I.S.); 2 Department of Functional Genomics and Experimental Pathology, Research Center for Functional Genomics, Biomedicine and Translational Medicine, “Iuliu Hatieganu” University of Medicine and Pharmacy, Str. V. Babes, No. 8, 400000 Cluj-Napoca, Romania; braicucornelia@yahoo.com (C.B.); ioananeagoe29@gmail.com (I.B.N.); 3 Department of Chemistry, Lensfield Road, Centre for Molecular Science Informatics, University of Cambridge, Cambridge CB2 1EW, UK; pruteanulavinia@gmail.com; 4 MEDFUTURE-Research Center for Advanced Medicine, “Iuliu Hatieganu” University of Medicine and Pharmacy, 23 Marinescu Street, 400015 Cluj-Napoca, Romania; 5 Department of Functional Genomics and Experimental Pathology, The Oncology Institute “Prof. Dr. Ion Chiricuta”, Republicii 34 Street, 400015 Cluj-Napoca, Romania; 6 Department of Food Science, University of Agricultural Sciences and Veterinary Medicine, Calea Manastur, No. 3-5, 400372 Cluj-Napoca, Romania; dan.vodnar@usamvcluj.ro

**Keywords:** intestinal porcine epithelial cells (IPEC-1), lactobacilli, microarray, genome, IPA analysis

## Abstract

Background: Food and feed supplements containing microorganisms with probiotic potential are of increasing interest due to their healthy promoting effect on human and animals. Their mechanism of action is still unknown. Using a microarray approach, the aim of this study was to investigate the differences in genome-wide gene expression induced by a mixture of three *Lactobacillus* strains (*L. rhamnosus*, *L. plantarum*, and *L. paracasei*) in intestinal porcine epithelial cells (IPEC-1) and to identify the genes and pathways involved in intestinal barrier functions. Methods: Undifferentiated IPEC-1 cells seeded at a density of 2.0 × 10^5^/mL in 24-wells culture plates were cultivated at 37 °C and 5% CO_2_ until they reached confluence (2–3 days). Confluent cells monolayer were then cultivated with 1 mL of fresh lactobacilli (LB) mixture suspension prepared for a concentration of approximately 3.3 × 10^7^ CFU/mL for each strain (1 × 10^8^ CFU/mL in total) for 3 h and analyzed by microarray using Gene Spring GX v.11.5. Results: The functional analysis showed that 1811 of the genes modulated by LB treatment are involved in signaling (95% up-regulation, 121 genes with a fold change higher than 10). The most enhanced expression was registered for *AXIN2* (axis inhibition protein 2-*AXIN2*) gene (13.93 Fc, *p* = 0.043), a negative regulator of β-catenin with a key role in human cancer. LB affected the cellular proliferation by increasing 10 times (Fc) the NF1 gene encoding for the neurofibromin protein, a tumor suppressor that prevent cells from uncontrolled proliferation. The induction of genes like *serpin peptidase inhibitor*, *clade A member 3* (*SERPINA 3*), *interleukin-20* (*IL-20*), *oncostatin M* *(OSM*), *granulocyte-macrophage colony-stimulating factor* (*GM-CSF*), and the suppression of *chemokine (C-X-C motif) ligand 2*/*macrophage inflammatory protein 2-alpha* (*CXCL-2/MIP-2*), *regulator of G-protein signaling 2* (*RGS2*), and of pro-inflammatory *interleukin-18* (*IL-18*) genes highlights the protective role of lactobacilli in epithelial barrier function against inflammation and in the activation of immune response. Conclusion: Gene overexpression was the predominant effect produced by lactobacilli treatment in IPEC-1 cells, genes related to signaling pathways being the most affected. The protective role of lactobacilli in epithelial barrier function against inflammation and in the activation of immune response was also noticed.

## 1. Introduction

Under normal conditions, the structure and functional integrity of the intestinal cell layer forms a physical and immunological barrier that prevents the access of foreign antigens including pathogens, food proteins, and toxins into the underlying tissues [1,2,3]. In this regard, intestinal epithelial cells are considered the “watchdogs” of the immune system [4]. They constitutively produce several cytokines and chemokines including transforming growth factor β (TGF-β) and interleukins (IL-1α, IL-6) which are crucial for the recruitment and activation of neutrophils, macrophages, dendritic cells, T and B cells, mucosal homeostasis, and cell growth [4,5]. Other cytokines such as interleukin-8 (IL-8), interleukin-1 β (IL-1β), and tumor necrosis factor-α (TNF-α) are also synthetized by normal epithelial cells and are markedly upregulated in response to microbial infection [6], where they contribute actively to the initiation of inflammatory cascade in the intestine.

Intestinal epithelial cells also have digestive and absorptive functions including the secretion of water and electrolytes to maintain the viscosity of the luminal content [7,8]. The digestive changes during the weaning period in which the mammal has to adapt to the solid diets and to withstand to pathogen challenge are stressful and interfere with intestinal development and function [9,10]. In the pig for example, post weaning anorexia alters gut integrity, one of the major aetiologic factors in gut associated disorders by increasing mucosal permeability, disturbance in nutrient and ion transport, and stimulation of inflammation at epithelium level [10,11]. In the last decade, many studies have tested probiotics for ameliorating inflammation and gastrointestinal disease symptoms and for decreasing pathogen load in animal [12]. It has been shown that probiotic bacteria reinforce and maintain the intestinal barrier by interaction with the cellular junctional complex (either adherence junction or tight junction) proteins and by up-regulating their expression [13]. Lactobacilli are among the most well-known and safest probiotics with significant beneficial effects on gut health [1,14,15,16]. They contribute to the maturation and modulation of the immune system by enhancing cytokines production, and inhibiting pathogenic bacterial adhesion and activity by lactic acid secretion [17]. Many studies report on the efficiency of lactobacilli in preventing the intestinal diseases, the ability to restore the normal microbiota and the prevention of inflammation [18,19]. These authors [18,19] showed that four distinct *Lactobacillus* spp. (*L. acidophilus*, *L. gasseri*, *L. fermentum*, and *L. rhamnosus*) reinforce the epithelial barrier via their effect on the expression and functionality of adherence junction protein E-cadherin and the influence is species specific [18]. Also, *L. sobrius* could inhibit the Enterotoxigenic *Escherichia coli* (ETEC) pathogen internalization by acting on tight junction and cytoskeleton protein [19], while *L. amylovorus*, a novel *lactobacillus* isolated from unweaned pigs and its cell free supernatant, counteract the ETEC action by suppressing the activation of the different steps of TLR4 signaling and the over-production of inflammatory cytokines IL-8 and IL-1β in pig explants and intestinal Caco-2 cell challenge by ETEC pathogen [20]. Conversely, experimental probiotics (*Lactobacillus* and *Bifidobacterium*) either reduced or did not affect the production of cytokines (IL-6, IL-12p70, and TNF-α) in epithelial and dendritic cell co-culture [21]. However, the majority of studies with lactobacilli has been focused on individual strains and their capacity to modulate the immune host response and to limit pathogen invasion [12,22] and very few investigated the effect of mixtures of different probiotic strains and their mechanism of action in pig. However, evidence showed that bacteria mixture can strengthen the effect of individual strains that might last longer.

Using a DNA microarray this study brings information on the differences in genome-wide gene expression induced by a mix of three *Lactobacillus* strains (*L. rhamnosus*, *L. plantarum*, and *L. paracasei*) in unchallenged intestinal porcine epithelial cells (IPEC-1) cultivated under normal functional conditions. Since probiotics are used as supplement not only for treatment but also for prevention it is important to know their effect in non-pathological challenged conditions in order to discover their mechanism of action and to increase their applications. The most genomic studies were oriented on the cross-talk between intestinal epithelial cells and probiotic microorganisms in response to pathogen bacteria.

The modulation of specific genes and pathways involved in intestinal barrier functions was also analyzed. Gene expression profile has proven to be a powerful tool in studying the molecular cross-talk between intestinal epithelial cells and microorganisms, probiotic bacteria included. Pig is a relevant human model due to its similarity to human’s genome and immune response to different environmental agents.

## 2. Results

### 2.1. Microarray Screening

Porcine (*S. Scrofa*) V2 Genome microarray was used to identify the differences in genes expression in IPEC-1 cells treated or not with a mixture of three lactobacilli for 3 h. The mixture of lactobacilli used in this study contained *L. plantarum* (ATCC 8014, isolated from horse feces), strain included in the EFSA list of safe substances (QPS) with EFSA recognized probiotic properties, as well as *L. paracasei* (ATCC 335, isolated from food) and *L. rhamnosus* (IBNA02, isolated from food), whose probiotic potential was described in the literature. Data analysis was performed using GeneSpring GX Version 12.6.1 Software analysis. A total number of 13,950 genes were found to be differentially expressed when compared to control genes activation (over expression) being the predominant effect produced by LB treatment (12,678 up-regulated and 1272 down-regulated) considering a fold change of 2 and a *p*-value ≤ 0.05.

### 2.2. Functional Classification of Differentially Expressed Genes

Genes that were found to be significantly modulated by LB treatment were then subjected to a cluster analysis and classified into nine functional categories and pathways: signaling, cell signaling, proliferation, transcription factors, growth factors, cytokines, interleukins, immune response, and inflammatory response (Table 1). Within these clusters, the gene over-expression was seen to be the most significant effect of LB. The functional analysis showed that 1811 of the genes modulated by LB treatment are involved in signaling. From these, 1735 genes were up-regulated, 121 of them had a fold change greater than 4; the most enhanced expression was registered for *AXIN2* (axis inhibition protein 2-AXIN2) gene (13.93 Fc, *p* = 0.043). Seventy-six out of the 1811 genes were found to be down-regulated, expression of *RGS2* and *OR1L8* gene being the most suppressed: −6.67 Fc, *p* = 0.017 and −5.26 Fc, *p* = 0.259, respectively (Table 2).

The cluster of genes related to proliferation also encountered a high number of differentially expressed genes (692 genes): of these, 632/692 were up-regulated (with 59/632 exceeding a fold change of 4, e.g., *NF1*, 10.20 Fc) and 60/692 were down-regulated (*AREG*, -3.85 Fc, *IL1α*, -3.33 Fc, Table 3). Following this, was the cluster relating to transcription factors genes with 657 modulated genes, of which 597 were over expressed (the most up-regulated being, for instance, *TSHZ2*, 12.21 Fc, *p* = 0.058, *NF1*, 10.20 Fc, *p* = 0.133 and EMX1, 9.00 Fc, respectively) and 60 were suppressed (*PKNOX2*, −8.33 Fc, *p* = 0.015, Table 4).

LB treatment also enhanced the expression of genes encoding for growth factors. This category encountered 411 modified genes of which 398 genes were up-regulated (*HSPB1*, 7.67 Fc and *PHLPP1*, 7.89 Fc for example) and 13 genes were down-regulated (*IL1α*, −3.36 Fc).

In IPEC-1 cells, LB mixture modulated a lower number (between 100–200) of genes encoding for cytokines, interleukins, immune and inflammatory response and of these most were up-regulated (cytokines 224 genes, interleukins 186 genes, immune response 119 genes, and inflammatory response 205 genes). LB treatment up-regulated the expression of *SERPINA-3* (9.85-Fc), *IL-20* (6.23 Fc), *OSM* (6.19 Fc), *GM-CSF* (6.06 Fc) and suppressed that of *CXCL-2* (*MIP-2*, −6.25 Fc) and *RGS2* (−6.67 Fc) genes and of *IL-18* (−3.45 Fc) pro-inflammatory cytokine (Table 4 and Table 5).

### 2.3. Real-Time Quantitative Real Time Validation

**Figure 1:**
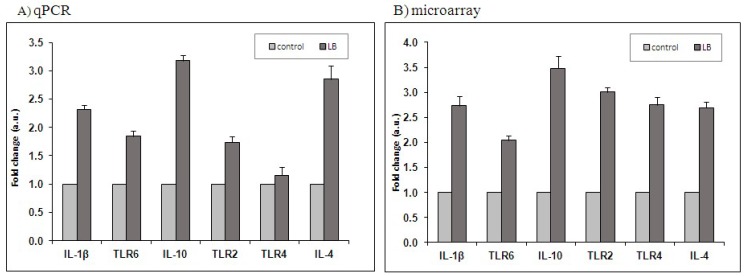
Validation of microarray results by real-time RT-PCR. Gene expression of six selected genes obtained by qPCR (**A**) was compared with that obtained by microarray (**B**). Each experiment was repeated four times (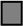) lactobacilli mixture versus (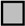) Control cells); *n* = 4.

Quantitative Real Time (qPCR) analysis for the expression of 6 genes (*IL-1β*, *TLR-6*, *IL-10*, *TLR-2*, *TLR-4*, *IL-4*) was used to validate the microarray results. The expression levels of selected genes showed a similar pattern (up-regulation) either in microarray (*IL-1β*, 2.73 Fc; *TLR-6*, 2.04 Fc; *TLR-2*, 3.01 Fc; *TLR-4*, 2.75 Fc; *IL-10*, 3.48 Fc; *IL-4*, 2.69 Fc) or in qPCR (*IL-1β*, 2.32 Fc; *TLR-6*, 1.84 Fc; *TLR-2*, 1.74 Fc; *IL-10*, 3.18 Fc; *IL-4*, 2.85 Fc) analysis (Figure 1). These results reveal a good correlation among the microarray and qRT-PCR data, being able to eliminate all the variability that can affect the quantification of the gene expression level.

### 2.4. Ingenuity Pathway Analyses

The results from Table 6, Table 7, Table 8 and Table 9 were obtained by using Ingenuity Pathway Analysis (IPA) and emphases that in IPEC-1 cells lactobacilli treatment could modulate to a different extent many genes involved in important canonical pathways, biological functions, and networks. Top canonical pathways such as Wnt/β-catenin and molecular mechanism of cancer (Table 6) as well as cellular functions like, cellular growth and proliferation, cell development, cell death and survival (598, 572 and 478 focus transcripts, Table 7 and Table 9) were modulated. IPA analyses demonstrated also the association of lactobacilli treatment with important diseases states and biological function such as Gastrointestinal Diseases (761 molecules), cancer (849 molecules), and inflammatory response (362 molecules), as shown in Table 8.

Several relevant biological networks, Cell-to-Cell Signaling and Interaction, (52 focus molecules), Cellular Development, Digestive System, (41 focus molecules), and cellular movement, (37 focus molecules) were also modulated by lactobacilli mixture (Table 9).

**Figure 2:**
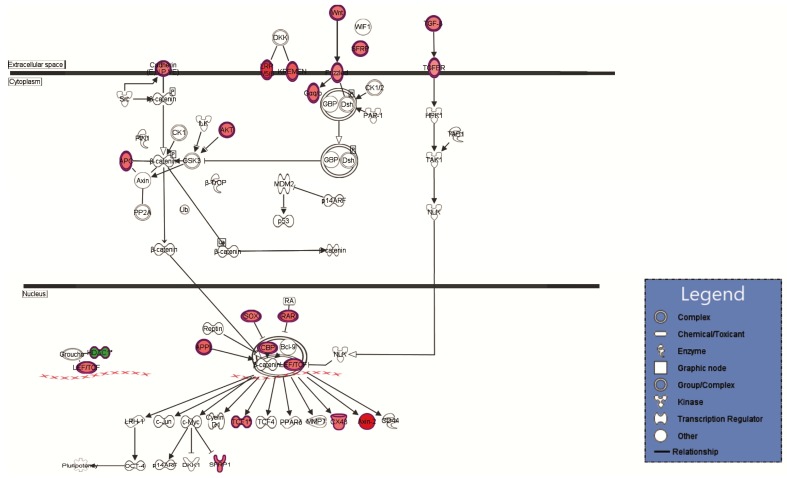
The predicted molecular connections between the differentially regulated genes in the extracellular space, cytoplasm, and nucleus of IPEC-1 cells. Information about the regulation of genes is included in the figure: the red color gradient from dark to light shows the degree of genes up-regulated in lactobacilli treated IPEC cells versus control cells; the green color shows the down-regulated in lactobacilli treated IPEC cells versus control cells; arrow (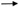) = direct relationship; (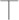) = inhibition.

**Figure 3:**
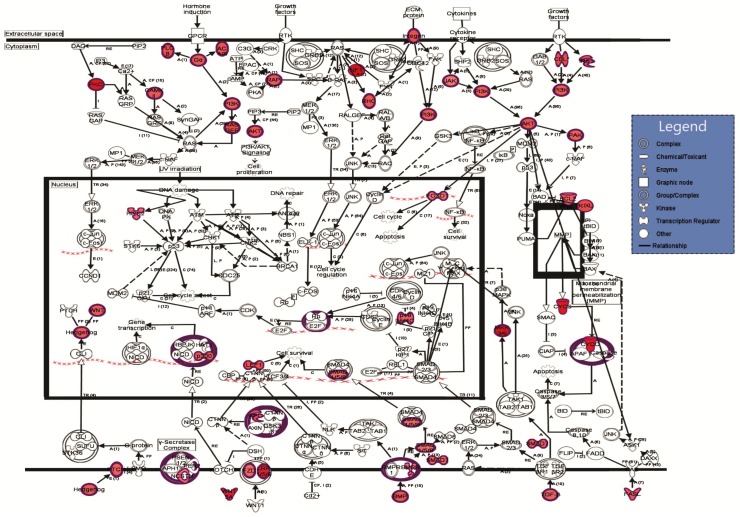
The predicted molecular connections between the differentially regulated genes in the extracellular space, cytoplasm, and nucleus of IPEC-1 cells under the LB mixture action. Information about the regulation of genes is included in the figure: the red color gradient from dark to light shows the degree of genes up-regulated in lactobacilli treated IPEC cells versus control cells. Nodes were used to connect the regulated genes; grey lines show inconsistent or no predicted effects; (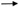) = direct relationship; (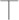) = inhibition; *n* = 4.

The networks from Figure 2 and Figure 3 showed a high number of predicted molecular interactions between the regulated genes indicating an up-regulation tendency (red color) in both cytoplasm and nucleus after the lactobacilli treatment.

## 3. Discussion

The normal functionality of the intestinal epithelial cells that cover the intestinal villi is essential for nutrient absorption [23]. Changes in the diet can affect its activity. Probiotic bacteria are often administered in order to confer health benefits to the intestinal epithelium such as cell proliferation and survival, prevention of epithelial injury, and improvement of epithelial barrier and immune function [24]. Probiotic applications are used therapeutically to prevent and treat gastrointestinal diseases including inflammatory bowel diseases, diarrhoea, gastroenteritis, irritable bowel syndrome, etc. [25,26], and a number of studies have elucidated some aspects of the mechanism involved in their effects.

Using a genome microarray analysis, we showed in this study that the exposure to lactobacilli mixture distinctly modulated a high number of genes encoding for different processes. The three *Lactobacilli* sp. were chosen as they are part of a probiotic bacterial product currently under development in the Institute of Biology and Animal Nutrition (IBNA) biotechnology laboratory, which has proved healthy benefits and excellent efficiency in reducing the gastrointestinal infections in pig as well as in other farm animals in experimental trails. A preliminary in vivo study of our team using the mixture reported a beneficial effect on both humoral (increase in IgG concentration) as well as on cellular immune response (decrease in cytokine TNF-α and increase in chemokine IL-8) [27]. Up-regulation of gene expression was the most prominent effect (with 15 genes’ expression being up-regulated by more than 10-fold). The genes failing within the signaling functional cluster were the most affected by lactobacilli mixture, followed by proliferation and transcription factors. A recent microarray study in neonatal gnobiotic pig using *Lactobacillus* rhamnosus and *Lactobacillus* acidophilus showed a similar alteration of canonical pathways involving genes associated with cell signaling and cellular growth and proliferation [11]. Likewise, in the study herein the IPA top canonical pathway analyses revealed that exposure to lactobacilli mixture significantly affected the Wnt/β-catenin signaling and Molecular mechanisms of cancer pathways (*p* = 1.32 × 10^−18^ and 4.29 × 10^−20^ respectively). Wnt/β-catenin signaling is an important pathway involved in development, cellular proliferation, and differentiation [28]. In the gastrointestinal tract Wnt/β-catenin pathway maintains the self-renewal capacity of epithelial stem cells and reestablishes the architecture of crypts after mucosal injury [29]. Microarray results of Hummel et al. [18] indicated that lactobacilli improved epithelial barrier function of T84 cells via enhancement of β-catenin phosphorylation, a subunit of the cadherin protein complex and an intracellular signal transducer in the Wnt signaling pathway with a key role in the creation and maintenance of epithelial cell layers and barrier through the regulation of cell growth and adhesion [30]. Mutation or overexpression of β-catenin is associated with many cancers, colorectal carcinoma among them [31]. It has been demonstrated that β-catenin is clearly involved in human cancer [32] together with other downstream components of Wnt pathway, adenomatous polyposis coli (APC), Dishevelled (Dsh), and the serine/threonine kinase GSK-3beta (GSK-3β) intracellular signaling molecules [32]. For example, inactivation of APC gene or activation of β-catenin mutation was found in more than 90% of all tumors; a hyper-activation of Wnt/β-catenin pathway leading to pathological transformation of gastrointestinal epithelium has been observed in the absence of a negative feedback mechanism that involve the up-regulation of *AXIN1* or *AXIN2* genes, negative regulators of β-catenin [29,33,34]. A former study [35,36] demonstrated that the overexpression of AXIN2 induced a dramatic reduction of β-catenin level in human colorectal cancer cell line SW480. In this regard, a recent study of Du et al. (2016) demonstrated that genistein-27, a derivative of isoflavone genistein was able to inhibit the proliferation of human colorectal cancer cells by reducing β-catenin nuclear localization and by increasing the expression of AXIN2 and APC (adenomatous polyposis coli) [37]. In the study herein, the microarray results showed that incubation of IPEC cells with lactobacilli mixture enhanced in a distinct manner the expression of AXIN2 (13.92 Fc).

The study of Taherian-Esfahani et al., 2016 [38] found that the expression of secreted frizzled related protein 2 (*SFRP2*) gene, another antagonist of Wnt pathway was increased in HT-29 cells after *L. rhamnosus* GG treatment and in HeLa cells after the treatment with supernatant of *L. crispatus* and *L. rhamnosus* GG culture. The IPA top network from Figure 2 also illustrates that APC, SFRP, AXIN2, and other key components involved in Wnt signaling transduction system were positively regulated whereas histone deacetylase (*HDAC*) gene was negatively regulated. The HDAC3 expression decreased after a dietary treatment with a broccoli sprout extract supplement [39]. Many studies showed that the uses of lactobacilli are promising tools against the uncontrolled proliferation occurred in cancer [40]. Supernatants and lactobacilli extract reduced cell proliferation, cell migration, invasion, and increased apoptosis of colorectal tumor cell CaCO-2 and HT-29 [40,41,42]. The results of our wide-genome study (Table 2, Figure 3) indicated a 10-fold increase of *NF1* gene expression encoding for neurofibromin protein which act as a tumor suppressor that prevent cells from growing and dividing too rapidly or in an uncontrolled way. NF1 is a negative regulator of the *ras* signal transduction pathway that stimulates cell division and growth. Patients with NF1 mutation are predisposed to benign as well as malignant tumors like neurofibroma and malignant peripheral nerve-sheath tumors [43]. It is believed that neurofibromatosis type 1, associated with an increased risk of gastrointestinal stromal tumors (GISTs) is caused by functionally biallelic losses of the tumor suppressor gene, *NF1* [43,44].

Probiotics have often been investigated for their protective effect against colitis and other pro-inflammatory gastrointestinal diseases. The recent study of Kumar et al. [11] reported that a probiotic mixture (VSL#3, manufactured in India by Sun Pharmaceutical Ind. Ltd.) reduced basal levels of pro-inflammatory cytokines and chemokines like monocyte chemotactic protein-2 (MCP-2), chemokine (C-X-C motif) ligand 2 (CXCL2) and improved epithelial barrier function in mucin deficient (Muc2^−/−^) animals or DSS-induced colitis. Likewise, assessment of anti-inflammatory activities of lactic acid bacteria in porcine intestinal epithelial (PIE) cells challenged with heat-killed enterotoxigenic *E. coli* showed a significant down-regulation in the levels of pro-inflammatory interleukins (IL-8, IL-6), and chemokines (MCP-1) [45]. Our study shows that when the three lactobacilli strains acted together the expression of genes associated with “inflammatory response” functional category was modified. For example, a strong down regulation (*p* < 0.002) of pro-inflammatory CXCL2 (MCP-1) chemokine was found (−6.25 Fc); the IPA Top Diseases and Bio Function analysis showed also a significant modulation of 362 molecules involved in inflammatory response. This reduction effect is most likely to be due to the regulation of RGS2 gene expression. In the present study, RGS2 gene expression was strongly down-regulated (−6.67 Fc) in the intestinal cells incubated with lactobacilli mixture. The RGS2 gene encodes for the RGS2 protein (regulator of G-protein signaling 2 protein), which is known to have putative tumor suppression function by inhibiting the proliferation and migration of cancer cells through the acceleration of GTP-ase activity and controlling the alpha subunits of G proteins [46]. In accordance with this observation, Boelte et al. (2011) [47] reported that genetic deletion or inactivation of RGS2 results in a significant reduction of tumor growth and of MCP-1 in a Rgs2^−/−^ tumor myeloid derived suppressor cells (MDSCs) mouse model. While searching for molecular mediators responsible for tumor derived MDSCs, they found an increased level of RGS2 in tumors. When MCP-1 was blocked with a neutralizing antibody it resulted in diminished tumor metastases. These findings suggest that the expression of RGS is different in various types of cancers [46].Our results have confirmed also that administration of lactobacilli could have implications beyond the treatment of cancer, bowel diseases, etc. The interest in using probiotic bacteria against intestinal inflammatory response has been increased lately. The up-regulation of genes like SERPINA 3 (by 9.85-fold), IL-20 (by 6.23-fold), OSM (by 6.19-fold), and the down-regulation of the previously mentioned CXCL-2 which regulates cell proliferation and differentiation during inflammation highlight the protective role of lactobacilli in epidermal barrier function against inflammation. 

Findings have shown that probiotics like lactobacilli could be associated with beneficial modulation of immune response. In our study, for instance, the three lactobacilli mixture enhanced expression of GM-CSF (granulocyte-macrophage colony-stimulating factor gene) and PBD-2 (beta-defensin 2) and suppressed expression of IL-18 pro-inflammatory cytokine genes. Indeed, recent studies showed that probiotics, lactobacilli among them activate both natural and acquired immune responses by induction of a broad array of cytokines (IL-1β, IL-10, IL-8, MIP-1α, MIP-1β, and GM-CSF) as well as by suppression of others (i.g. IL-1α, TNF-α) [48,49,50]. 

The extrapolation of pig genes to human resulted in an average overlapping yield of approximately 60% compared to 3.64 when applying to other methods.

## 4. Materials and Methods

### 4.1. IPEC-1 Cell Culture

The Intestinal Porcine Epithelial Cell line-1 (IPEC-1) was used in this study as previously described [51]. Briefly, cells were grown at 37 °C, under 5% CO_2_ atmosphere incubation in complete Dulbecco’s Modified Eagle Medium DMEM/F-12 medium (Sigma, Saint Louis, MO, USA), supplemented with antibiotics, 5% foetal bovine serum (Sigma), 2 mM l-glutamine, 15 mM Hepes (Sigma), epidermal growth factor, 5 µg/L (Sigma), insulin (10 µg/mL), transferrin (5 µg/mL), and sodium selenite (5 ng/mL) (ITS Premix, Sigma).

### 4.2. Lactobacilli (LB) Preparation

Three lactic acid bacteria, Lactobacillus plantarum (ATCC8014, isolated from horse feces), *Lactobacillus* paracasei (ATCC335, isolated from food; provided by from National Research Institute Cantacusino, Bucharest, Romania, Cantacusino Collection, ID12353 and ID13239), and *Lactobacillus* rhamnosus (IBNA collection, IBNA02, isolated from food) kept in glycerol were cultured individually in anaerobic plastic tubs in DeMan Rogosa and Sharpe (MRS, Sigma) medium (1:10), overnight at 37 °C under anaerobic conditions as described by [19]. The following day the culture was diluted 1:15 in fresh MRS and cultivated another 3–4 h until the OD600 reached values of about 1 ± 0.1, which corresponded to 1.0 × 10^9^ CFU/mL for *L. plantarum*, 2.9 × 10^8^ CFU/mL for *L. paracasei* and 3.2 × 10^8^ CFU/mL for *L. rhamnosus*. Bacterial viability was tested by CFU counts after agar plating of bacterial serial dilution. Lactic bacteria were harvested by centrifugation at 4000 rpm for 10 min at 4 °C, and resuspended in antibiotic free DMEM/F-12 medium (Sigma). The lactobacilli mixture suspension was prepared in IPEC cell culture medium for a concentration of approximately 0.5 × 10^8^ CFU/mL for each strain (1.5 × 10^8^ CFU/mL in total) and added to the epithelial cells.

### 4.3. Bacterial Treatment

Undifferentiated IPEC-1 cells were seeded at a density of 2.0 × 10^5^/mL in 24-well cell culture plates (Costar, Corning, NY, USA) and cultivated at 37 °C and 5% CO_2_ until they reached confluence (2–3 days). Then, the cell culture supernatant was removed and 1 mL of fresh lactobacilli mixture suspension with 1 × 10^8^ CFU/mL prepared as describe above was added to the IPEC-1 monolayer and incubated for 3 h at 37 °C and 5% CO_2_. After incubation and removal of supernatant the cells were washed with PBS, resuspended in 0.5 mL of Trizol (Sigma) and stored at −80 °C until analyses. The experiments were carried out in four independent replicates.

### 4.4. Extraction of Total RNA for Microarray

The total RNA from untreated and LB treated IPEC-cells was isolated using a QiagenRNeasy midi kit (QIAGEN GmbH, Hilden, Germany), according to the manufacturer’s recommendations as described by [52] and their quality and integrity was verified by using an Agilent 2100 bioanalyzer and Agilent RNA 6000 nano kit (Agilent Technologies, Santa Clara, CA, USA). The RNA integrity number (RIN) score ranged between 8 and 10. The purified RNA samples were preserved at −80 °C until used.

### 4.5. Microarray Assay

Agilent SurePrint G3 Custom Gene Expression Microarray 8 × 60K slides (Santa Clara, CA, USA) were used for hybridization, the array consisting in 60 mer oligonucleotide probes with a total number of 45,220 features and 1417 Agilent features control. 200 ng of purified RNAs was tagged using Low Input Quick Amp Labeling Kit, Two-Color (48 rxn, 5190-2306, Santa Clara, CA, USA), that implies reverse transcribing the mRNA in the presence of T7-oligo-dT primer to furnish cDNA and afterwards in vitro transcribing with T7 RNA polymerase in the presence of Cy3-CTP and Cy5-CTP to construct labelled cRNA. The labelled cRNA of the LB-treated and the control specimens from every biological replicate were labelled with Cy-3 and Cy-5 dyes and hybridized in duplicate with dye reversal to the Agilent Whole Genome Porcine (S. Scrofa)- V2 8 × 60K microarray Slides (G2519F-026440, Santa Clara, CA, USA) according to the Agilent manufacturer’s protocol. The slides were washed with Agilent buffer and scanned on Agilent Microarray Scanner G2565BA (Santa Clara, CA, USA) at high and low resolution. 

### 4.6. Statistical Analyses of Microarray Data

The pre-processing, normalization, and differential analysis of data were done as described by [53] by using the Gene-Spring GX version 12.6.1 software (Santa Clara, CA, USA). Smoothing adjustment in the differences of Cy5 intensity was done by using Lowes normalization which removes such variation. Student’s *t*-test were applied in order to assess the statistical differences in gene expression, the data are presented as log2 value transformed in fold change by applying the function of Power (2, log2-Value).

Genes with fold change of ±2 and a *p*-value of ≤0.05 were considered statistically significant using Gene-Spring GX version 12.6.1 Software. The genes expression significantly modulated by the treatment was subjected to a cluster analysis based on Pearson coefficient correlation algorithm and classified into nine functional categories and pathways (signaling, cell signaling, proliferation, transcription factors, growth factors, cytokines, interleukins, immune response, and inflammatory response) to assess the global effect on biological processes. All the transcript sequences of *Sus scrofa* were extrapolated to their human counterparts using Homology Based Annotation retrieved from NCBI Database (available online: www.ncbi.nlm.nih.gov) and BLAST [46]. Ingenuity Pathway Analysis (IPA; available online: http://www.ingenuity.com) was applied in order to evaluate the impact of lactobacilli on human health. A network interaction between the genes with an altered expression level is also presented in order to better understand the LB mode of action in the context of functional clusters and pathway.

### 4.7. Validation of Gene Expression by Quantitative Real-Time PCR (RT-qPCR)

For the validation of microarray analysis, the expression levels of five randomly selected genes (IL-1β, TLR6, TLR4, TRL2, IL-10) were measured by real time RT-qPCR in all samples used for microarray analysis as described by [51]. Primer pairs used for validation are listed in Table 10.

## 5. Conclusions

Gene overexpreession was the predominant effect produced by lactobacilli treatment in IPEC-1 cells, genes related to signaling pathways being the most affected (95% up-regulation, 121 genes with a fold change higher than 10). lactobacilli mixture significantly affected the Wnt/β-catenin signaling by up-regulating AXIN2 gene (13.93-fold), a negative regulator of β-catenin, which plays a key role in human cancer as well as the cellular proliferation by increasing 10 times (Fc) the NF1 gene encoding for the neurofibromin protein, a tumour suppressor that prevent cells from uncontrolled proliferation. The induction of genes like SERPINA 3, IL-20, OSM, and GM-CSF, and the suppression of CXCL-2 (MIP-2), RGS2 genes, and pro-inflammatory IL-18 cytokine highlight the protective role of lactobacilli in epithelial barrier function against inflammation and in the activation of immune response. However, further studies are needed to confirm these results and to understand the in vivo underlying mechanism of lactobacilli action. 

## Figures and Tables

**Table 1:** Functional classification of differentially expressed genes under lactobacilli mixture treatment.

Function Name	LB Treatment				
	Up-Regulated		Down-Regulated		Total Found Genes
	No.	%	No.	%	No.
Signaling	1735	95.8	76	4.2	1811
Cell signaling	132	96.4	5	3.7	137
Proliferation	632	91.3	60	8.7	692
Transcription factors	597	90.9	60	9.1	657
Growth factors	398	96.8	13	3.2	411
Cytokines	224	95.7	10	4.3	234
Interleukin	186	93.0	14	7.0	200
Immune response	110	92.4	9	7.6	119
Inflammatory response	205	94.0	13	6.0	218

**Table 2:** List of the most strongly upregulated or downregulated genes involved in signaling in IPEC-cells.

Gene ID	Gene Symbol	Gene Description	Fold Change	Regulation
		Signaling		
GACC01000361	*nf1*	neurofibromin 1 (NF1), transcript variant 1	10.20	up
AK349266	*fuz*	fuzzy homolog (Drosophila) (FUZ), transcript variant 1	10.63	up
-	*-*	-	10.70	up
XM-001928433	*or2m3*	olfactory receptor, family 2, subfamily M, member 3 (OR2M3) ectonucleoside triphosphate	11.63	up
AK345382	*entpd1*	diphosphohydrolase 1 (ENTPD1), transcript variant 1	12.13	up
-	*-*	-	13.45	up
XM-003482962	*axin2*	axin 2	13.93	up
AB530146	*rgs2*	regulator of G-protein signaling 2, 24 kDa	−6.67	down
XM-001925049	*or1l8*	olfactory receptor, family 1, subfamily L, member 8	−5.26	down
-	*-*	-	−4.17	down
NM-001001861	*cxcl2*	chemokine (C-X-C motif) ligand 2	−4.00	down
NM-214376	*areg*	amphiregulin	−3.85	down
NM-214376	*areg*	amphiregulin	−3.85	down
AY609724	*tcf21*	transcription factor 21	−3.70	down

**Table 3:** List of the most strongly upregulated or downregulated genes involved in proliferation in IPEC-cells.

Gene ID	Gene Symbol	Gene Description	Fold Change	Regulation
		Proliferation		
GACC01000361	*nf1*	neurofibromin 1 (NF1), transcript variant 1	10.20	up
-	*-*	-	10.70	up
-	*-*	-	13.45	up
XM-003482962	*axin2*	axin 2	13.93	up
NM-214376	*areg*	amphiregulin	−3.85	down
-	*-*	-	−3.45	down
NM-214029	*il1α*	interleukin 1, alpha (IL1α)	−3.33	down
AY610314	*ube2v2*	ubiquitin-conjugating enzyme E2 variant 2	−3.33	down

**Table 4:** List of the most strongly upregulated or downregulated genes associated with transcription factors and inflammatory response in IPEC-cells.

Gene ID	Gene Symbol	Gene Description	Fold Change	Regulation
		**Transcription Factors**		
XM-003125031	*emx1*	empty spiracles homeobox 1 (EMX1)	9.00	up
GACC01000361	*nf1*	neurofibromin 1 (NF1), transcript variant 1	10.20	up
AK347929	*tshz2*	teashirt zinc finger homeobox 2 (TSHZ2), transcript variant 1	12.21	up
XM-003361490	*pknox2*	PBX/knotted 1 homeobox 2	−8.33	down
AY609724	*tcf21*	ref|Homo sapiens transcription factor 21 (TCF21), transcript variant 2	−3.70	down
		**Inflammatory Response**		
XM-003131278	*prkcα*	protein kinase C, alpha	5.13	up
-	*-*	-	5.58	up
AK396677	*pla2g7*	phospholipase A2, group VII (platelet-activating factor acetylhydrolase, plasma)	5.82	up
XM-001929161	*osm*	oncostatin M	6.19	up
XM-003130465	*il20*	interleukin 20	6.23	up
AY669080	*bmp2*	bone morphogenetic protein 2	6.87	up
AK232615	*serpina3*	serpin peptidase inhibitor, clade A (alpha-1 antiproteinase, antitrypsin), member 3	9.85	up
AK345252	*cxcl2*	chemokine (C-X-C motif) ligand 2	−6.25	down
XM-003129107	*cxcl2*	chemokine (C-X-C motif) ligand 2	−6.25	down
XM-003126166	*cxcl2*	chemokine (C-X-C motif) ligand 2	−4.00	down
NM-001001861	*cxcl2*	chemokine (C-X-C motif) ligand 2	−3.45	down
-	*-*	-	−3.45	down
AY577905	*cxcl2*	chemokine (C-X-C motif) ligand 2	−3.45	down
NM_214029	*il1α*	interleukin 1, alpha	−3.33	down

**Table 5:** List of the most strongly upregulated or downregulated genes involved in immune response in IPEC-cells.

Gene ID	Gene Symbol	Gene Description	Fold Change	Regulation
		Immune Response		
NM-001102680	*cd1d*	CD1d molecule (CD1D)	5.21	up
AY506573	*pbd-2*	beta-defensin 2 mRNA, complete cds	5.24	up
D21074	*csf2*	GM-CSF for granulocyte-macrophage colony-stimulating factor, complete cds	6.06	up
XM-003129107	*loc100525528*	hypothetical protein LOC100525528 (LOC100525528)	−6.25	down
NM-001001861	*cxcl2*	chemokine (C-X-C motif) ligand 2 (CXCL2)	−4.00	down
U68701	*Il-18*	interleukin-18, complete cds	−3.45	down
AY577905	*cxcl2*	CXCL2, complete cds	−3.45	down
NM-214029	*Il-1a*	interleukin 1, alpha (IL1α)	−3.33	down

**Table 6:** Top gene Canonical Pathways associated with Lactobacilli treatment—Ingenuity Pathway Analysis (IPA).

Name	LB Treatment	
	*p*-Value	Ratio
Role of Macrophages, Fibroblasts and Endothelial Cells in Rheumatoid Arthritis	9.33 × 10^−21^	56/309 (0.181)
Molecular mechanism of cancer	4.29 × 10^−20^	61/374 (0.163)
Wnt/β-catenin	1.32 × 10^−18^	39/169 (0.231)
Human embryonic Stem Cell Pluripotency	1.33 × 10^−18^	36/143 (0.252)

**Table 7:** Top gene molecular and cellular functions associated with lactobacilli treatment—IPA analysis.

Name	LB Treatment	
	*p*-Value	Molecules
Cellular growth and proliferation	2.79 × 10^−20^–1.53 × 10^−124^	598
Cellular Development	2.79 × 10^−20^–1.13 × 10^−117^	572
Gene Expression	1.83 × 10^−33^–1.33 × 10^−117^	439
Cellular Movement	3.35 × 10^−20^–2.04 × 10^−95^	392
Cell death and Survival	4.95 × 10^−20^–5.24 × 10^−86^	478

**Table 8:** Top gene Diseases and Bio Function associated with lactobacilli treatment—IPA analysis.

Name	LB Treatment	
	*p*-Value	Molecules
Cancer	2.41 × 10^−20^–1.11 × 10^−58^	849
Organism Injury and Abnormalities	2.41 × 10^−20^–1.11 × 10^−58^	860
Gastrointestinal Diseases	2.38 × 10^−20^–1.53 × 10^−42^	761
Developmental Disorder	3.43 × 10^−21^–5.85 × 10^−42^	282
Inflammatory Response	5.00 × 10^−33^–2.08 × 10^−43^	362

**Table 9:** Top gene networks associated with lactobacilli treatment—IPA analysis.

Associated Network Function	Score
Cell Signaling Cell-to-Cell Signaling and Interaction, Cell Cycle	52
Gene Expression, Cellular Development, Digestive System Development and Function	41
Gene Expression, Skeletal and Muscular Disorders, Skeletal and Muscular System Development and Function	37
Cellular Movement, Hematological System Development and Function, Immune Trafficking	37
Gene Expression, Hematological System Development and Function, Tissue Morphology	37

**Table 10:** Oligonucleotide Polymerase chain reaction (PCR) primers.

Gene	Accesion No.	Primer Source	Primer Sequence (5′→3′)	Orientation	Tm (°C)	Amplicon Lenght (bp)	References
IL-1β	NM_214055	Pig	ATGCTGAAGGCTCTCCACCTC	forward	62	89	[54]
			TTGTTGCTATCATCTCCTTGCAC	reverse	59		
TLR2	NM_213761.1	Pig	TCACTTGTCTAACTTATCATCCTCTTG	forward	59	162	[55]
			TCAGCGAAGGTGTCATTATTGC	reverse	59		
TLR4	NM_001113039.1	Pig	GCCATCGCTGCTAACATCATC	forward	60	108	[55]
			CTCATACTCAAAGATACACCATCGG	reverse	59		
TLR6	NM_213760.1	Pig	AACCTACTGTCATAAGCCTTCATTC	forward	59	95	[55]
			GTCTACCACAAATTCACTTTCTTCAG	reverse	59		
IL-10	NM_214041.1	Pig	GGCCCAGTGAAGAGTTTCTTTC	forward	54	55	[56]
			CAACAAGTCGCCCATCTGGT	reverse	51		
IL-4	NM_214123.1	Pig	CAACCCTGGTCTGCTTACTG	Forward	52	173	[57]
			CTTCTCCGTCGTGTTCTCTG	reversed	52		
Cyclophilin A	NM_214353.1	Pig	CCCACCGTCTTCTTCGACAT	forward	54	92	[58]
			TCTGCTGTCTTTGGAACTTTGTCT	reverse	55		
β-actina	NM_213978.1	Pig	GGACTTCGAGCAGGAGATGG	forward	60	230	[59]
			GCACCGTGTTTGCGTAGAGG	reverse	62		
